# Lack of an effect of nephron-specific deletion of adenylyl cyclase 3 on renal sodium and water excretion or arterial pressure

**DOI:** 10.14814/phy2.12316

**Published:** 2015-03-06

**Authors:** Wararat Kittikulsuth, Deborah Stuart, Alfred N Van Hoek, Donald E Kohan

**Affiliations:** Division of Nephrology, University of Utah Health Sciences Center, Salt Lake City Veterans Affairs Medical CenterSalt Lake City, UT

**Keywords:** Adenylyl cyclase 3, blood pressure, nephron, sodium excretion, water excretion

## Abstract

Adenylyl cyclase (AC)-stimulated cAMP plays a key role in modulating transport and channel activity along the nephron. However, the role of individual adenylyl cyclase isoforms in such regulation is largely unknown. Since adenylyl cyclase 3 (AC3) is expressed throughout nephron, we investigated its role in the physiologic regulation of renal Na^+^ and water transport. Mice were generated with inducible nephron knockout of AC3 (AC3 KO) by breeding mice with loxP-flanked critical exons in the *Adcy3* gene with mice expressing Pax8-rtTA and LC-1 transgenes. After doxycycline treatment at 1 month of age, nephron AC3 KO mice had 100% *Adcy3* gene recombination in all renal tubule segments, but not in glomeruli. Sodium intake, urinary Na^+^ excretion, glomerular filtration rate, and blood pressure were similar between nephron KO mice and the controls during normal, high, and low Na^+^ diets. Plasma renin concentration was not different between the two groups during varied Na^+^ intake. Moreover, there were no differences in urine volume and urine osmolality between the two genotypes during normal or restricted water intake. In conclusion, these data suggested that AC3 is not involved in the physiological regulation of nephron Na^+^ and water handling.

## Introduction

Adenylyl cyclases (ACs) are a family of enzymes that catalyze the conversion of ATP into cAMP. Cyclic AMP plays a key role in modulating nephron transport and channel activity in response to hormones including vasopressin (Schafer and Troutman 1990; Wallace et al. 2002), glucagon (Bailly et al. 1985; Chabardès et al. 1996), parathyroid hormone (Chabardès et al. 1975; Bailly et al. 1985; Friedman et al. 1996, 1999), calcitonin (Chabardès et al. 1976; Bouley et al. 2011) and others. AC isoforms 3, 6, and 9 are expressed throughout the nephron, including proximal tubule (PT), thick ascending limb (TAL), distal convoluted tubule (DCT), and collecting duct (CD) (Rieg and Kohan 2014). Mice lacking AC6 have increased urine output and decreased urine osmolality with reduced AQP-2 and phosphorylation of AQP2 (Rieg et al. 2010). Furthermore, AC6-deficient mice have reduced responsiveness to ddAVP-induced Na^+^-K^+^-2Cl^−^ cotransporter expression and phosphorylation as well as phosphorylation of the NaCl cotransporter (Rieg et al. 2013). Our group demonstrated that mice with CD-specific knockout of AC6 (CD AC6 KO) had a urinary concentrating defect and lacked AVP-stimulated epithelial Na^+^ channel activity (Roos et al. 2012, 2013). While the role of AC6 in the nephron has been investigated, the role of AC3 in nephron water and Na^+^ handling has focused only on the CD. CD AC3 KO mice have similar urine volume, urine osmolality and urinary Na^+^ excretion as compared with control mice (Kittikulsuth et al. 2014). However, mice with global knockout of AC3 tended to have increased Na^+^ and water excretion in the face of reduced glomerular filtration rate (GFR) (Pluznick et al. 2009). Taken together, these data raise the possibility that AC3 in the nephron, perhaps in segments upstream of the CD, modulates renal Na^+^ and water excretion. Consequently, the goal of this study was to investigate the role of AC3 in the nephron on blood pressure and renal Na^+^ and water excretion.

## Methods

### Animal study approval

All animal use and welfare adhered to the NIH Guide for the Care and Use of Laboratory Animals following protocol reviews and approval by the Institutional Laboratory Animal Care and Use Committee of the University of Utah Health Sciences Center.

### Generation of inducible whole nephron-specific adenylyl cyclase 3 knockout mice

**Figure 1:**
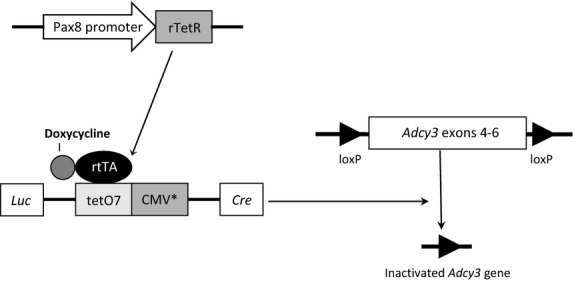
Schema for generation of inducible whole nephron-specific adenylyl cyclase 3 knockout mice.

Mice transgenic for Pax8-rtTA and LC-1 transgenes were bred with mice containing loxP-flanked (floxed) exons 4–6 of the *Adcy 3* gene (Kittikulsuth et al. 2014) to yield inducible whole nephron-specific AC3 mice that were homozygous for the floxed *Adcy 3* gene and hemizygous for the Pax8-rtTA and LC-1 transgenes (Fig.1). As previously described (Traykova-Brauch et al. 2008; Stuart et al. 2012), the Pax8-rtTA transgene contains 4.3> kb of the 5′ untranslated region along with exon 1, intron 1, exon 2, and part of intron 2 of the *Pax8* gene driving expression of the reverse tetracycline transactivator. The LC-1 transgene encodes tetracycline-inducible bicistronic Cre recombinase and luciferase (Traykova-Brauch et al. 2008; Stuart et al. 2012). To obtain mice lacking *Adcy 3* gene in the nephron (nephron AC3 KO), 1-month-old male mice that were homozygous for the floxed *Adcy 3* gene and hemizygous for Pax8-rtTA and for LC-1 transgenes were given 2> mg/ml doxycycline (DOX) in 2% sucrose drinking water for 14 days, followed by at least 21> days off DOX before conducting experiments. A similar protocol of DOX treatment was also used in control littermates that were homozygous for the floxed *Adcy 3* gene and hemizygous for LC-1, but lacked Pax8-rtTA (so DOX cannot induce gene recombination). All mice were on a C57/BL6 background that had been backcrossed for at least 10 generations.

### Genotyping

Tail DNA from nephron AC3 KO and control littermate mice was PCR amplified with the following primers: AC3F 5′-ctgctttgtcattacaatttcc-3′ and AC3R 5′-tgaggactgcctttctagag-3′ which yield a 275 bp product from the floxed *Adcy3* gene and a 241 bp product from the wild-type allele (Kittikulsuth et al. 2014). Transgenes were identified using the following primers: Pax8-rtTAF 5′-ccatgtctagactggacaaga-3′ and Pax8-rtTAR 5′-catcaatgtatcttatcatgtctgg-3′ which yields a 600> bp product; and LC-1F 5′-tcgctgcattaccggtcgatgc-3′ and LC-1R 5′-ccatgagtgaacgaacctggtcg-3′ which yields a 480> bp product (Stuart et al. 2012).

### Analysis of Adcy3 gene recombination

Brain, heart, lung, liver, spleen, intestine, and kidneys from nephron AC3 KO mice were excised. One kidney was separated into renal cortex and papilla. Another kidney was cut longitudinally into sections containing the entire corticomedullary axis. The kidney sections were incubated with Hanks Balanced Salt Solution (HBSS) containing 2 mg/ml collagenase and 2 mg/ml hyaluronidase for 20 min at 37°C. The incubated tissue was rinsed with HBSS and stored on ice until dissection of the tubules. Dissection of glomeruli, proximal tubules, thick ascending limb, distal convoluted tubules, and cortical and inner medullary collecting ducts was performed at 4°C. DNA from selected organs and microdissected tubules was isolated and PCR amplified to evaluate for *Adcy3* gene recombination using primers spanning exons 4–6 of the *Adcy3* gene: F 5′-caggtagaattcttgctggttc-3′ and R 5′-tgaggactgcctttctagag-3^′^. Recombination of the *Adcy3* gene yields a 400 bp product; the size of the unrecombined *Adcy3* gene is ∽2000 bp.

### Analysis of mRNA

RNA from renal papilla from nephron AC3 KO and control mice was extracted using guanidinium isothiocyanate and acid phenol, reverse transcribed, and cDNA levels determined for AC3 and GAPDH using real-time PCR (StepOne Plus, Applied Biosystems, Foster City, CA). PCR was performed according to instructions provided by the manufacturer using the Taqman Gene Expression Assay (Applied Biosystems) with Adcy3 (Cat.# Mm00460371_m1) and GAPDH (Cat.# Mm99999915_g1) primers.

### Metabolic cage studies

For water handling studies, nephron AC3 KO and control mice were placed in metabolic cages and given 9 ml of a gelled diet made from 62 g of PMI rodent powdered diet (LD101; LabDiet, Richmond, Indiana), 7 g gelatin, and 110 ml water with free access to drinking water on day 1 (baseline). Then, mice were placed on water restriction by given 9 ml gelled diet made from 248 g of PMI rodent powdered diet, 7 g gelatin, and 110 ml water for 2 days with no access to drinking water. In all studies, urine was analyzed for volume and osmolality.

For Na^+^ balance studies, nephron AC3 KO and control mice were fed a normal (0.3%) Na^+^ diet for 6 days, followed by a high (3.15%) Na^+^ diet for 7 days, and then followed by a low (0.01%) Na^+^ diet for 7 days. The diets consisted of the normal water gelled diet above with NaCl content modified. At the end of each diet, blood was taken from the tail vein for determination of plasma renin concentration (PRC). Twenty-four-hours urine collection was done on each day of all diets and was analyzed for volume and Na^+^.

### Blood pressure monitoring and glomerular filtration rate determination

Blood pressure was monitored in nephron AC3 KO and control mice by radiotelemetry (TA11-PAC10; Data Sciences International, St. Paul, MN) with catheters inserted into the right carotid artery. The mice were allowed to recover for 1 week after surgery. Blood pressure and heart rate were monitored daily during normal (3 days), high (7 days), and low (7 days) Na^+^ intake.

Glomerular filtration rate (GFR) was determined in nephron AC3 KO and control mice on a normal Na^+^ diet. The NIC-Kidney (Mannheim Pharma & Diagnostics GmbH, Mannheim, Germany) was attached to the shaved back and fluorescence determined for 15 min. Subsequently, 7.5 mg/100 g body weight FITC-sinistrin (Mannheim Pharma) was injected retro-orbitally and fluorescence determined over the next hour. The kinetics of fluorescence decay was used to calculate GFR per manufacturer software.

### Plasma and urine analysis

Blood was collected in mice on a normal Na diet (*n* = 5/group) and plasma analyzed for creatinine and blood urea nitrogen (BUN) using a commercially available quantitative colorimetric analysis according to the manufacturer's protocol (Quantichrom, BioAssay Systems, Hayward, CA). PRC was measured by enzyme immunoassay as the amount of angiotensin I (AngI) generated after incubation with excess angiotensinogen (Peninsula Laboratories, San Carlos, CA) and expressed as the amount of AngI generated per hour per ml of plasma. Urine Na^+^ was analyzed using an Easylyte Analyzer (Medica, Bedford, MA). Urine osmolality was determined by freezing point depression (Osmett II, Precision System, Natick, MA).

### Statistical analysis and data presentation

Data are reported as mean ± standard error of the mean. Studies involving varying Na^+^ and water intakes and hemodynamics were analyzed by analysis of variance. Plasma creatinine, blood urea nitrogen (BUN), PRC, and mRNA studies were analyzed by a two-sided unpaired Student's *t*-test. The criterion for significance was *P *≤* *0.05.

## Results

### Confirmation of inducible whole nephron AC3 KO

**Figure 2:**
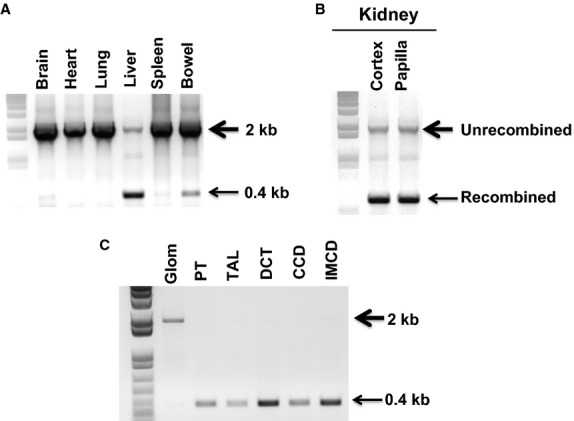
Representative blot of *Adcy3* gene DNA recombination in nephron AC3 KO mice from organ panel (A), renal cortex and medulla (B), and microdissected glomeruli and tubules (C) (proximal tubule; PT, thick ascending limb; TAL, distal convoluted tubule; DCT, cortical collecting duct; CCD, inner medullary collecting duct; IMCD). The 2 kb band is the unrecombined allele and the 0.4 kb band is the recombined allele (a representative blot is shown for *n* = 3 mice/group).

Mice which were heterozygous for Pax8-rtTA and LC-1 transgenes and homozygous for the floxed *Adcy3* gene were born at the expected frequency and showed no developmental abnormalities. These mice lived up to at least 6 months of age and had no apparent histologic renal abnormalities. In addition, no renal histologic abnormalities were apparent up to 6 months after DOX treatment. PCR of DNA from brain, heart, lung, and spleen from nephron AC3 KO mice showed no recombination of the *Adcy3* gene, however, DNA recombination occurred in liver, renal cortex, and renal medulla with a small amount of recombination in intestine (Fig.2). To confirm that DNA recombination occurred only in renal tubules, microdissected proximal tubules (PT), thick ascending limbs (TAL), distal convoluted tubules (DCT), and cortical (CCD) and inner medullary collecting ducts (IMCD) and glomeruli from nephron AC3 KO mice were examined. *Adcy3* gene recombination, with no apparent unrecombined DNA, was found in all renal tubule segments examined (PT, TAL, DCT, OMCD, and IMCD), but not in glomeruli (Fig.2).

**Figure 3:**
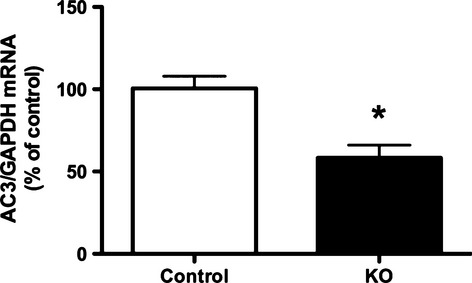
Relative mRNA expression of AC3 in the inner medulla from nephron AC3 KO mice and the controls. *P < 0.05 versus floxed control (*n* = 6 mice/group).

AC3 mRNA expression was assessed in kidney using real-time PCR. AC3 mRNA in microdissected PT, TAL, DCT, or CD from control mice was very low, requiring over 40 cycles to detect, while GAPDH was detected after 20 cycles. Consequently, differences between nephron AC3 KO and control AC3 mRNA in renal tubules could not be reliably ascertained. However, PCR of inner medulla using primers that span exons 11–12 in the *Adcy3* gene revealed that nephron AC3 KO mice had a 50% reduction in AC3 mRNA content as compared to control mice (Fig.3). Given that AC3 is also expressed in non-IMCD cell type in the inner medulla (Hoffert et al. 2005; Uawithya et al. 2008), the unrecombined band for AC3 in papilla and inner medulla could represent a contribution from interstitial cells, vasa recta and/or thin limbs of Henle. PCR of cortex did not consistently show differences in AC3 mRNA, however, glomeruli contained significant AC3 mRNA, which obviated seeing differences in AC3 mRNA levels in tubules between the two genotypes. Finally, immunostaining of whole kidney or western analysis of renal cortex or medulla did not reveal clear staining or bands for AC3 despite trying several different commercially available AC3 antibodies with varied antigen retrieval protocols (the latter for staining); this is a relatively common problem with detecting AC isoforms which have a very high degree of amino acid sequence homology between one another. Hence, to the best of our ability, and largely based on the DNA recombination data, it appears that AC3 was effectively targeted in the nephron.

### Effect of nephron AC3 KO on blood pressure and Na^+^ handling

**Figure 4:**
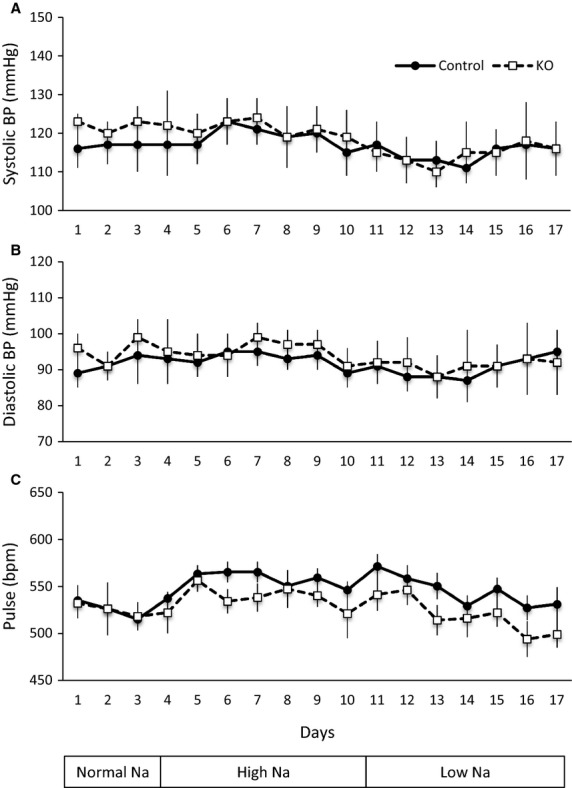
Effect of nephron AC3 KO on systolic BP (A), diastolic BP (B), and heart rate (C) during normal (0.3%), high (4%), or low (0.01%) Na^+^ diets (*n* = 5 mice/group).

Systolic blood pressure (SBP), diastolic blood pressure (DBP), and pulse pressure were determined by telemetric system during normal, high, and low Na^+^ intake in nephron AC3 KO and control littermates (Fig.4). There were no differences in SBP, DBP, and pulse pressure on any of the days on different level of Na^+^ intakes. There were also no differences in SBP, DBP, or pulse pressure comparing daytime or night time values between genotypes.

**Figure 5:**
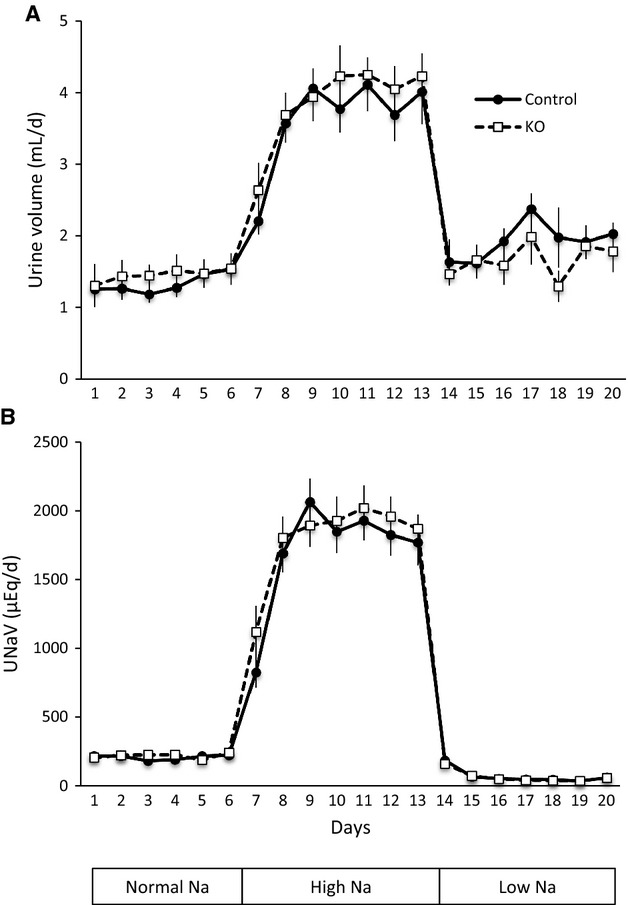
Effect of nephron AC3 KO on urine volume (A) and urinary Na^+^ excretion (B) during normal (0.3%), high (4%), or low (0.01%) Na^+^ diets (*n* = 9–10 mice/group).

Nephron AC3 KO and control littermates were placed on a normal Na^+^ diet for 6 days, switched to a high salt diet for 7 days, followed by a low salt diet for 7 days. Urine volume and urinary Na^+^ excretion were similar between nephron AC3 KO and the controls on any of the days during different levels of Na^+^ intake (Fig.5). Food intake and water intake were similar between the two groups during normal, high, and low Na^+^ intake.

### Effects of nephron AC3 KO on plasma renin concentration

**Figure 6:**
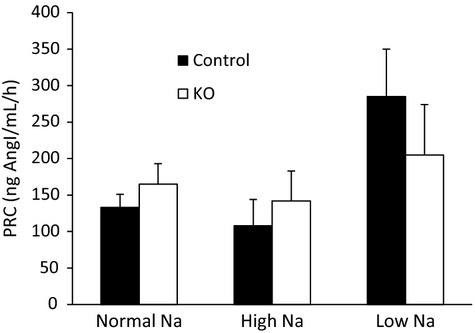
Effect of nephron AC3 KO on plasma renin concentration during normal, high, and low Na^+^ intake (*n* = 9–10 mice/group).

As compared to a normal diet, PRC was increased in both nephron AC3 KO mice and the controls fed a low Na^+^ diet. There was no difference in PRC between the two genotypes during normal, high, or low Na^+^ feeding (Fig.6).

### Effects of nephron AC3 KO on water handling

**Figure 7:**
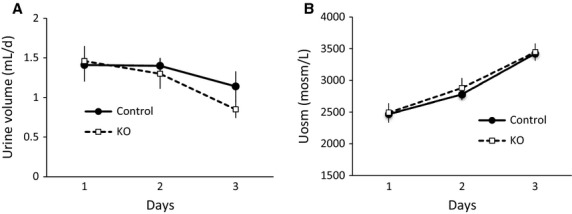
Effect of nephron AC3 KO on urine volume (A) and urine osmolality (B) during normal water intake followed by 2 days of water restriction (*n* = 9–10 mice/group).

Nephron AC3 KO and control mice were placed on a gel diet with free water access for 1 day followed by water restriction for 2 days. Water intake, urine volume, and urine osmolality were similar between nephron AC3 KO mice and the controls during normal water intake. There were no differences in urine volume and urine osmolality between the two genotypes during 2-day water restriction (Fig.7).

### Effect of nephron AC3 KO on GFR

During a normal Na diet, plasma creatinine was similar between the controls and nephron AC3 KO mice (0.186 ± 0.015 vs. 0.187 ± 0.017 mg/dl, respectively). Moreover, BUN was comparable between the two groups (21 ± 1 mg/dl for controls vs. 20 ± 1 mg/dl for nephron AC3 KO). To more directly assess GFR, mice were administered FITC-sinistrin and fluorescence kinetics determined using the NIC-Kidney; control and nephron AC3 KO mice had similar values (358 ± 10 *μ*l/min in control and 369 ± 8 *μ*l/min in nephron AC3 KO mice, *N* = 5 each group).

## Discussion

The major findings of the current study are that absence of AC3 in the nephron has no effect on: 1) urinary Na^+^ excretion and blood pressure during normal, high, or low Na^+^ intake; 2) PRC during varied Na^+^ intake; or 3) urine volume and urine osmolality during water restriction. Notably, no effect of AC3 KO on GFR was observed. These results suggest that AC3 is not involved in the physiological regulation of nephron Na^+^ and water handling.

Given our negative results, a valid question is whether it was reasonable to hypothesize that AC3 might modulate nephron Na^+^ and/or water excretion. Several considerations supported the possibility that nephron AC3 could be involved. First, mice with global AC3 deficiency have reduced GFR (0.19 ± 0.04 ml/min in AC3^−/−^ mice as compared to 0.31 ± 0.02 in wild-type controls), however, Na^+^ excretion tended to be increased in AC3-deficient mice (0.39 ± 0.14 mEq/min/100 g BW in AC3^−/−^ mice as compared to 0.23 ± 0.05 mEq/min/100 g BW in wild-type controls) (Pluznick et al. 2009). The AC3^−/−^ mice weighed ∽30% more than wild-type animals; since Na^+^ excretion was factored for BW, this means that despite having a lower GFR, absolute Na^+^ excretion uncorrected for BW was 2.2-fold greater in AC3^−/−^ mice as compared to controls. While the validity of such analysis can be questioned, these studies at least raised the possibility that nephron AC3 may regulate renal Na^+^ excretion. Second, previous work by our group found no evidence for a role for CD principal cell AC3 in control of blood pressure or renal Na^+^ or water excretion, however, angiotensin II-induced cAMP accumulation in inner medullary CD from CD AC3 KO mice was reduced as compared to control mice (Kittikulsuth et al. 2014). Finally, several studies suggested, albeit through pharmacological approaches not specifically targeting a single AC isoform, that AC3 might be involved in control of renal water excretion (Ausiello and Hall 1981; Chou et al. 2000; Hoffert et al. 2005). Taken together, the above considerations provided reasonable justification for performance of the current study.

If nephron AC3 does not regulate renal Na^+^ and water excretion, what role might it serve? Consideration of studies on AC3 in the DCT suggests that this AC isoform may be involved in modulation of transport of ions other than Na^+^. AC3 is relatively highly expressed in the DCT as compared to other nephron segments (Pluznick et al. 2009; Kittikulsuth et al. 2014); this nephron segment reabsorbs Na^+^, Mg^2+^, and Ca^2+^ (Voets et al. 2004; Boros et al. 2009; Houillier 2014) through cAMP-regulated pathways (Friedman et al. 1996; Gesek and White 1997; Dai et al. 1998, 2001). Olfactory G protein (Golf), which is activated by olfactory receptors (OR), is uniquely localized within the nephron to the DCT and macula densa (Pluznick et al. 2009); Golf is specifically associated with activation of AC3 (DeMaria and Ngai 2010). Since multiple ORs are expressed throughout the kidney, including in the DCT (Pluznick et al. 2009; Huling et al. 2012; Rajkumar et al. 2014), the possibility is raised that a novel regulatory mechanism involving AC3 exists in the DCT that senses various chemicals and ultimately modulates ion transport therein. Examination of this system is clearly indicated, albeit well beyond the scope of the current study.

The current study potentially has limitations. To the best of our ability, we believe that nephron AC3 was completely deleted, however, it is possible that a small fraction of AC3 remains. While it seems highly unlikely that such a tiny residual of AC3 could matter, one must consider that other AC isoforms could compensate for absence of AC3. This is a very difficult issue to address since it essentially requires codeletion of two or more AC isoforms; such analysis would likely best be done, at least at first, in cell culture models and would still be open to questions about physiological relevance. Another possibility is that other physiological regulatory systems could be masking an effect of nephron AC3 deletion, including changes in renal nerve activity, any number of circulating factors, or local autocrine or paracrine systems. Such changes cannot be excluded, however, the lack of any alteration in PRC, which is normally very sensitive to alterations in blood pressure and alterations in renal Na^+^ excretion, suggests that no major direct effect of nephron AC3 deletion on renal Na^+^ excretion occurred.

In summary, the present study demonstrates that nephron AC3 KO mice do not have altered BP or renal Na^+^ and water excretion under varying Na^+^ or water intake conditions. Further studies are required to examine the role of other nephron AC isoforms in the control of nephron Na^+^ and water transport. In addition, studies are required for examining nephron AC3 modulation of renal handling of other electrolytes.
